# Implantation of two second‐generation trabecular micro‐bypass stents and topical travoprost in open‐angle glaucoma not controlled on two preoperative medications: 18‐month follow‐up

**DOI:** 10.1111/ceo.12958

**Published:** 2017-06-02

**Authors:** John Berdahl, Lilit Voskanyan, Jonathan S Myers, Dana M Hornbeak, Jane Ellen Giamporcaro, L Jay Katz, Thomas W Samuelson

**Affiliations:** ^1^ Vance Thompson Vision Sioux Falls South Dakota USA; ^2^ S.V. Malayan Ophthalmology Centre Yerevan Armenia; ^3^ Wills Eye Hospital Jefferson Medical College Philadelphia Pennsylvania USA; ^4^ Glaukos Corporation San Clemente California USA; ^5^ Minnesota Eye Consultants Minneapolis Minnesota USA

**Keywords:** bypass, glaucoma, IOP, MIGS, trabecular

## Abstract

**Importance:**

Additional data are sought regarding treatment options for glaucoma, a major cause of global blindness.

**Background:**

The study assessed outcomes following standalone implantation of two second‐generation trabecular micro‐bypass stents and postoperative topical prostaglandin in eyes with open‐angle glaucoma not controlled on two preoperative medications.

**Design:**

The study design is a prospective, nonrandomized, open‐label study at a tertiary‐care ophthalmology centre.

**Participants:**

Subjects had open‐angle glaucoma with preoperative intraocular pressure of 18–30 mmHg on two medications, a medication washout phase, and post‐washout intraocular pressure of 22–38 mmHg. All subjects (N = 53) have been followed for 18 months.

**Methods:**

One day following implantation of two second‐generation trabecular micro‐bypass stents, subjects started topical travoprost. Medication washout was repeated at month 12.

**Main Outcome Measures:**

The main outcome measure was the proportion of eyes with intraocular pressure reduction ≥ 20% versus medicated baseline intraocular pressure with reduction of one medication at 12 months.

**Results:**

At 12 months, 91% of eyes achieved intraocular pressure reduction ≥ 20% with reduction of one medication. All eyes had intraocular pressure ≤ 18 mmHg with reduction of one medication, and 87% had intraocular pressure ≤ 15 mmHg. Mean intraocular pressure on one medication was ≤ 13.0 mmHg (≥ 34% reduction) through 18 months. Mean post‐washout intraocular pressure at month 13 was 33% lower than preoperative unmedicated intraocular pressure. No adverse events occurred through 18 months.

**Conclusions and Relevance:**

In open‐angle glaucoma eyes on two preoperative medications, treatment with two second‐generation trabecular stents and one postoperative prostaglandin resulted in mean intraocular pressure ≤ 13 mmHg with reduction of one medication, with favourable safety. These findings show the utility of second‐generation trabecular bypass with postoperative prostaglandin in patients with open‐angle glaucoma.

## Introduction

Glaucoma is a leading cause of blindness worldwide, and its management requires persistent life‐long therapy. The therapeutic goal of various treatments – such as medications, laser procedures, microinvasive glaucoma surgery (MIGS) or traditional filtering surgery – is to reduce intraocular pressure (IOP) in order to prevent damage to the optic nerve. Over the past decade, MIGS procedures with implantation of trabecular micro‐bypass stents have enabled IOP reduction via connecting the anterior chamber with Schlemm's canal, thereby bypassing the damaged trabecular meshwork. Numerous studies have assessed outcomes for up to 5 years following implantation of the first U.S. Food and Drug Administration‐approved trabecular micro‐bypass device, the iStent® Trabecular Micro‐Bypass (Glaukos Corporation – San Clemente, CA, USA). These data have demonstrated that implanting single^1^, ^2^, ^3^, ^4^ or multiple^5^, ^6^, ^7^, ^8^ first‐generation iStent devices, either with or without cataract surgery, can provide long‐term IOP and medication reduction in patients with mild to moderate glaucoma. In addition, MIGS procedures with these stents have demonstrated a positive benefit‐to‐risk profile in comparison to that of filtering surgeries^9^ or suprachoroidal stent placement.^10^, ^11^

In addition to the body of literature on this first‐generation stent, more recent studies have demonstrated similarly favourable outcomes with the implantation of two second‐generation iStent *inject* devices (iStent *inject*® Trabecular Micro‐Bypass, Glaukos Corporation – San Clemente, CA, USA) either with cataract surgery or in a standalone procedure.^12^, ^13^, ^14^, ^15^, ^16^ The MIGS Study Group, which was formed to study outcomes of standalone stent implantation in patients with various degrees of open‐angle glaucoma (OAG), previously reported substantial IOP and medication reduction through 36 months following implantation of two iStent devices and postoperative travoprost in eyes with OAG not controlled on two ocular hypotensive medications.^6^, ^7^ These findings reflected the beneficial effects of two proven treatment modalities: conventional outflow via trabecular micro‐bypass and uveoscleral outflow via a prostaglandin analogue. In a similar study design, we assessed outcomes after standalone implantation of two second‐generation iStent *inject* devices and one postoperative prostaglandin in 53 subjects with OAG not controlled on two preoperative medications.

## Methods

Patients with OAG not controlled on two topical ocular hypotensive medications were enrolled over a 7‐month period into this prospective, open‐label, single‐arm interventional study. Patients were required to present with cup‐to‐disc (C:D) ratio ≤ 0.9, IOP 18 and 30 mmHg, normal anterior chamber angle by gonioscopy, and best‐corrected visual acuity (BCVA) better than or equal to 20/100. Patients were excluded if they had any prior glaucoma surgery except iridotomy, or if they had uveitic, traumatic, neovascular, angle‐closure or vascular‐disease‐associated glaucoma. Following a medication washout period of either 5 days (carbonic anhydrase inhibitors), 2 weeks (alpha agonists) or 4 weeks (beta blockers and prostaglandin analogues), preoperative IOP was required to be between 22 and 38 mmHg to further qualify patients. Preoperative and postoperative assessments included IOP measurement, BCVA slit lamp and fundus examinations, C:D ratio, central corneal thickness, visual field, medication use, and ocular complications and interventions. Subjects were evaluated at postoperative day 1; week 1; months 1, 3, 6 and 12; and planned for every 6 months thereafter until month 60. Medication washout was performed at each annual visit, followed by a visit 1 month later to measure IOP and resume travoprost. If postoperative IOP exceeded 21 mmHg at any postoperative visit, additional ocular hypotensive medication was to be started.

This single‐site study was conducted at the S.V. Malayan Ophthalmological Centre in Yerevan, Armenia. Surgeons included one staff surgeon (L.V.) and 10 visiting surgeons from the MIGS Study Group (Appendix 1). Glaucoma‐trained ophthalmologists at the Centre completed all preoperative and postoperative examinations. Subjects were examined preoperatively and postoperatively by glaucoma‐trained staff ophthalmologists. The study was performed according to the international guidelines ICH‐GCP and ISO EN 14155:2011 and the tenets of the Declaration of Helsinki. Subjects signed an informed consent in order to join the study, and ethics committee approval was obtained before study initiation. The registration number for this study is NCT02873806 (www.clinicaltrials.gov).

**Figure 1:**
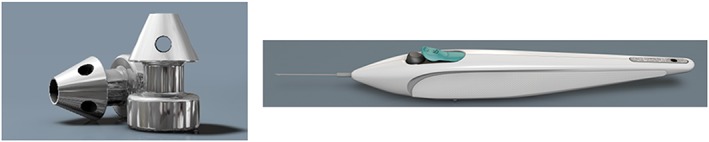
The iStent *inject* and iStent *inject* Trabecular Micro‐Bypass Stent System.

Prior studies have described the second‐generation iStent *inject* device and implantation technique (Fig. 1).^12^, ^13^, ^14^, ^15^ In this technique, the surgeon advances a single disposable stainless steel inserter through a temporal clear corneal incision, then implants *ab internally* two pre‐loaded stents into the nasal Schlemm's canal at approximately two clock‐hours apart. Each single‐piece stent is a titanium, heparin‐coated device with dimensions of 360 μm length and 230 μm width, multiple lateral outlet lumens for fluid outflow, and a symmetric design that allows for implantation in either right or left eyes. The stents are designed to decrease IOP by enhancing natural trans‐trabecular outflow from the anterior chamber to Schlemm's canal. After surgery, patients received topical anti‐infective (for 1 week) and anti‐inflammatory (for 4 weeks) medications. Topical travoprost was started on postoperative day 1 and continued throughout follow‐up except during medication washouts. All medications were provided to patients free of charge throughout the study).

We performed proportional analyses on 12‐month findings of IOP reduction ≥ 20% from baseline unmedicated IOP, and IOP ≤ 18 mmHg and IOP ≤ 15 mmHg, in eyes on one medication. These analyses were repeated with 18‐month data. Preoperative and postoperative IOP also was assessed. We assessed safety variables including slit lamp and fundus examinations, adverse events and secondary surgical interventions, and BCVA. Descriptive statistics (mean and standard deviation) were computed for continuous variables.

## Results

### Subject accountability, demographics and pre‐study parameters

A total of 53 qualified subjects (51 phakic, two pseudophakic) underwent *ab interno* implantation of two iStent *inject* devices over a 7‐month period. In addition, two patients who did not meet screening criteria (due to having three preoperative medications) also underwent surgery, but their data were excluded from analysis. All subjects have been followed for 18 months, and follow‐up is ongoing; thus, findings presented in this paper reflect a snapshot in time of data available at the time of manuscript submission.

Demographic and preoperative parameters are shown in Table 1. Mean age was 64.7 ± 9.6 years, with approximately equal gender distribution. Preoperative mean medicated IOP was 19.7 ± 1.5 mmHg on two medications, and mean unmedicated IOP (after preoperative washout) was 24.9 ± 1.1 mmHg. For all but one subject, the two preoperative medications included a topical prostaglandin. All subjects were Caucasian.

**Table 1:** Subject demographic and preoperative characteristics (*N* = 53)

Total	*N* = 53
Age (years), mean ± SD	64.7 ± 9.6
Lens status, phakic/pseudophakic	51/2
Gender, male/female	27/26
Preoperative C:D ratio, mean ± SD	0.7 ± 0.1
Preoperative BCVA 20/40 or better, % (*n*)	87% (*n* = 46)
Preoperative medicated IOP (mmHg), mean ± SD	19.7 ± 1.5
Preoperative # medications, mean ± SD	2 ± 0
Proportion of eyes on medication classes	
Prostaglandin + beta‐blocker	62% (*n* = 33)
Prostaglandin + carbonic anhydrase inhibitor	36% (*n* = 19)
Beta‐Blocker + carbonic anhydrase inhibitor	2% (*n* = 1)
Preoperative post‐washout IOP (mmHg), mean ± SD	24.9 ± 1.1

BCVA, best‐corrected visual acuity; SD, standard deviation; C:D, cup‐to‐disc; IOP, intraocular pressure; mmHg, millimetres of mercury.

### Efficacy

**Figure 2:**
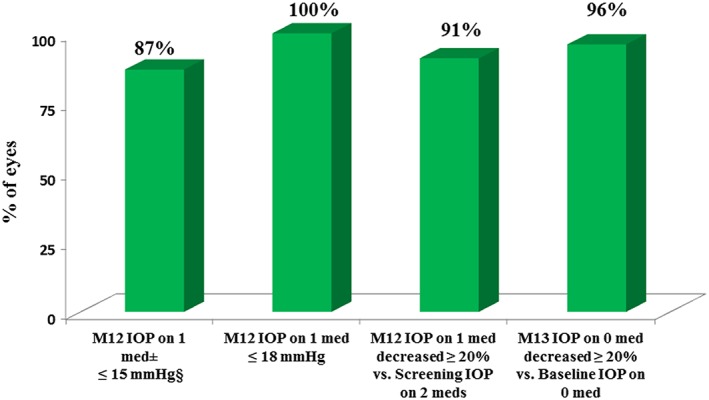
Proportional analysis of postoperative intraocular pressure (IOP) and medication usage (*N* = 53 at all time points). Med, medication; mmHg, millimetres of mercury.

**Figure 3:**
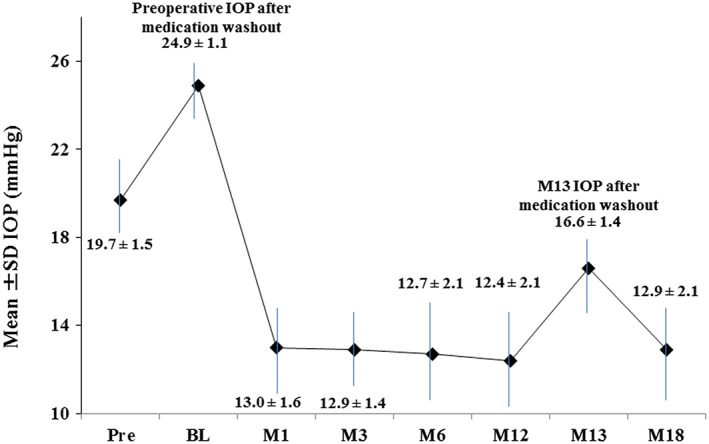
Mean intraocular pressure (IOP) over time (*N* = 53 at all time points). SD, standard deviation; mmHg, millimetres of mercury; Pre, preoperative; BL, baseline.

At 12 months postoperative, 91% of the 53 treated eyes had achieved an IOP reduction ≥ 20% from medicated baseline with reduction of one medication (Fig. 2). All eyes had IOP ≤ 18 mmHg, and 87% had IOP ≤ 15 mmHg with reduction of one medication (Fig. 2). Figure 3 shows the mean IOP over time for the 18‐month period after stent implantation. Mean medicated IOP was 13.0 mmHg or less (reduction of ≥ 34% from medicated preoperative mean IOP); 18‐month medicated IOP was 12.9 ± 2.1 mmHg, or 37% lower than preoperative mean IOP. Further, medication burden was halved to one medication through 18 months. At this exam, one subject was placed on one additional medication for IOP of 18 mmHg. The mean unmedicated (post‐washout) IOP decreased 33%, to 16.6 ± 1.4 mmHg at month 13 from 24.9 ± 1.1 mmHg preoperatively.

### Safety

**Figure 4:**
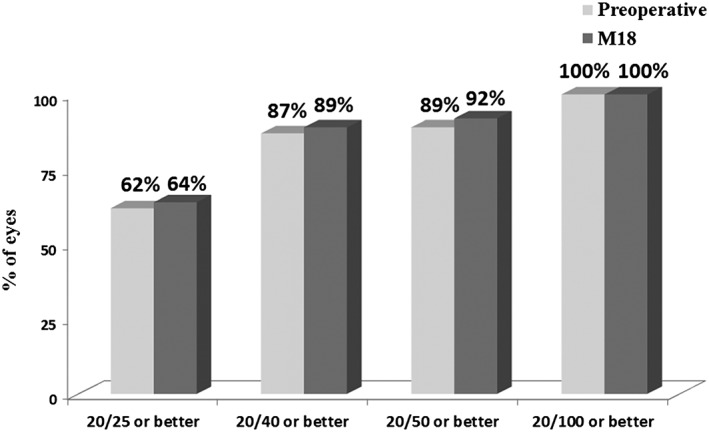
Preoperative and month 18 best‐corrected visual acuity (*N* = 53 at all time points).

All patients underwent uneventful implantation of two iStent *inject* devices in a standalone procedure, and no intraoperative adverse events occurred. No subjects experienced postoperative or device‐related adverse events through 18 months. BCVA, mean C:D ratio, visual field mean deviation and central corneal thickness were generally unchanged over time through month 18 (Fig. 4 and Table 2).

**Table 2:** Screening and postoperative mean cup‐to‐disc ratio, visual field, and central corneal thickness, available eyes at each visit†

	Screening	M6	M12	M18
*N*	53	53	53	37
C:D Ratio, mean (SD)	0.7 (0.1)	0.7 (0.1)	0.7 (0.1)	0.7 (0.1)
VF – mean deviation (dB), mean (SD)	−5.6 (5.4)	−5.3 (5.1)	−5.5 (5.5)	−5.5 (5.5)
VF – pattern standard deviation (dB), mean (SD)	3.8 (3.4)	3.6 (3.4)	3.7 (3.5)	3.7 (3.7)
Corneal thickness (μm), mean (SD)	538.8 (38.1)	540.6 (37.0)	540.2 (37.0)	539.7 (36.6)

† This constitutes only eyes that had measurements for all parameters at each time point. This includes *N* = 53 at screening, month 6 and month 12; and *N* = 37 at month 18.

C:D, cup‐to‐disc; dB, decibels; μm, micrometre; SD, standard deviation; VF, visual field.

## Discussion

The present study is the first to evaluate the performance of implantation of two second‐generation iStent *inject* devices as a sole procedure and one postoperative prostaglandin in subjects with OAG not controlled on two preoperative medications. This allows for an assessment of the combined effect of improving conventional outflow (via proven trabecular bypass stents) and uveoscleral outflow (via a prostaglandin analogue medication). Such a combination may optimize outcomes for patients on more than one medication who need more IOP‐lowering than that produced by either outflow pathway alone.

In this study, reductions in both mean IOP and medication burden were observed through 18 months postoperative: 12.9 ± 2.1 mmHg versus 19.7 ± 1.5 mmHg, and two versus one medication(s). Performance was favourable in regards to the primary and secondary efficacy endpoints, with 91% of eyes achieving an IOP reduction ≥ 20% versus baseline (100% had IOP ≤ 18 mmHg and 87% had IOP ≤ 15 mmHg) with reduction of one medication at 12 months. Subject accountability remained excellent at 100% through 18 months.

The significantly reduced mean IOP (≤ 13 mmHg through 18 months) supports the viability of using two second‐generation stents with postoperative prostaglandin to treat patients with inadequately controlled OAG and a moderate medication burden. The reduction in medications is particularly meaningful given the substantial cost of medications to glaucoma patients,^17^ who often are taking medication for at least one other chronic disease.^18^ Furthermore, the reduction from two to one medications is specifically valuable given the greatly improved patient adherence with single versus multiple eye drops.^19^

Regardless of number of medications, patient adherence to treatment regimens has long been documented to be low.^20^, ^21^ Thus, the study's measurement of preoperative and postoperative unmedicated IOP is clinically very valuable, as it reflects the effect of iStent *inject* alone, in the absence of medications or cataract surgery. For this measure, the mean unmedicated IOP decreased to 16.6 mmHg at month 13 versus 24.9 mmHg preoperatively, a reduction of 33%.

In addition to positive clinical outcomes, favourable safety was maintained over the 18 months of follow‐up, with no intraoperative, postoperative or device‐related adverse events. One additional medication was started in one eye at 18 months (IOP was 18 mmHg), but all remaining eyes remained on only travoprost. No eyes underwent secondary glaucoma or cataract surgery through 18 months. These favourable safety parameters are consistent with the many years of safety data that exist for trabecular stents and topical prostaglandin medications. Additionally, because no patients in this study required secondary surgery such as trabeculectomy or other filtering procedures during the 18‐month follow‐up period, this study suggests the potential to delay or prevent the need for such surgeries in uncontrolled OAG. Given the relatively high rate of adverse events observed with filtering surgery,^22^, ^23^ the benefit of this delay to patient quality of life may be substantial.

We have noted certain drawbacks in this study. Given its open‐label study design, neither clinicians nor subjects were masked to treatment. All subjects were Caucasian. No consistent preoperative lens grading system was employed. There was no sham surgical control group. Because stent implantation was completed as a standalone procedure, such a control group (which would undergo only injection and removal of viscoelastic) was not obtained because of ethical considerations. However as is common in many studies of standalone interventions, we viewed it as reasonable for patients' preoperative data to serve as their own control. Neither diurnal IOP nor baseline IOP across multiple time points was measured. The screening IOP was not measured at multiple time points, thus introducing the possibility of regression to the mean. Medication compliance may differ based on whether a subject is enrolled in a clinical trial. Together, these points could confound the determination of the true effect of preoperative medication. The study's completion at a single site prevents an analysis of potentially confounding site‐dependent effects; it also could account for the lower degree of measurement variability observed versus a multi‐centre study evaluation (because of IOP measurements by the same glaucoma‐trained study physicians, for example). The IOP requirements in this study also may have contributed to lower IOP variance. As per the study protocol, only descriptive statistics were employed; future work could consider adding non‐parametric tests or other inferential statistical tests. Lastly, this report covers data through 18 months, so future studies will be needed to assess outcomes after a longer follow‐up period.

In conclusion, this prospective, unmasked, single‐arm study provides meaningful data on the IOP‐lowering and medication‐lowering effects of standalone iStent *inject* implantation together with postoperative prostaglandin medication. The substantial decrease in medicated IOP appears to reflect the combined effects of increased trabecular and uveoscleral outflow via two proven modalities, trabecular stents and prostaglandins; importantly, this reduction occurred while also decreasing medication burden. In addition, the study's medication washouts offer an informative estimation of the independent performance of the iStent *inject* device. These data support the value of second‐generation trabecular bypass and postoperative prostaglandin in treating patients with OAG and a moderate preoperative medication burden.

## Supporting information


**Appendix 1:** List of Participating Microinvasive Glaucoma Surgery (MIGS) Study Group Surgeons.
