# Supplementary material for: Implantation of two second‐generation trabecular micro‐bypass stents and topical travoprost in open‐angle glaucoma not controlled on two preoperative medications: 18‐month follow‐up
Source: Clin Exp Ophthalmol. 2017 Jun 2;45(8):797–802. doi: 10.1111/ceo.12958 (PMC5724487; doi:10.1111/ceo.12958)
Supplement: Supplementary file 1 — Appendix 1: List of Participating Microinvasive Glaucoma Surgery (MIGS) Study Group Surgeons. [file CEO-45-797-s001.docx]

**Appendix 1:** List of Participating Microinvasive Glaucoma Surgery (MIGS) Study Group Surgeons

| **Surgeon Name** | **Affiliation** | **Study Participation** |
| --- | --- | --- |
| Iqbal Ike K. Ahmed, MD | University of Toronto  Toronto, Ontario, Canada | Visiting surgeon |
| John P. Berdahl, MD | Vance Thompson Vision  Sioux Falls, South Dakota, USA | Visiting surgeon |
| Richard Lewis, MD | Sacramento Eye Consultants  Sacramento, California, USA | Visiting surgeon |
| John A. Hovanesian, MD | Harvard Eye Associates  Laguna Hills, California, USA | Visiting surgeon |
| Jonathan S. Myers, MD | Wills Eye Hospital,  Philadelphia, Pennsylvania, USA | Visiting surgeon |
| Ilesh Patel, MD | Kirribilli, New South Wales, Australia | Visiting surgeon |
| Douglas J. Rhee, MD | University Hospitals / Case Medical Center  Cleveland, Ohio, USA | Visiting surgeon |
| Steven R. Sarkisian, Jr., MD | Dean A. McGee Eye Institute  Univ. of Oklahoma Health Science Center  Oklahoma City, Oklahoma, USA | Visiting surgeon |
| David Manning, MD | New South Wales, Australia | Visiting surgeon |
| Richard L. Lindstrom, MD | Founder and Attending Surgeon: Minnesota Eye Consultants  Adjunct Clinical Professor Emeritus:  Univ. of Minnesota Dept. of Ophth.  Minneapolis, Minnesota, USA | Visiting surgeon |
| Lilit Voskanyan, MD | S.V. Malayan Opthalmology Centre  Yerevan, Armenia | Staff surgeon |

Medical Monitor: L.J. Katz, MD, Wills Eye Hospital, Jefferson Medical College, Philadelphia, PA, USA. Email: [ljaykatz@gmail.com](mailto:ljaykatz@gmail.com)
